# Effects of Feeding Reduced Protein Diets on Milk Quality, Nitrogen Balance and Rumen Microbiota in Lactating Goats

**DOI:** 10.3390/ani15060769

**Published:** 2025-03-07

**Authors:** Runqi Fu, Ye Yu, Yuning Suo, Binlong Fu, Huan Gao, Lin Han, Jing Leng

**Affiliations:** 1 Yunnan Provincial Key Laboratory of Animal Nutrition and Feed, Yunnan Agricultural University, Kunming 650201, China; fandrunqi@163.com (R.F.); yy09091823@163.com (Y.Y.); 18313945858@163.com (Y.S.); binlongfu@126.com (B.F.); gaohuanhhhh@163.com (H.G.); 19286920591@163.com (L.H.); 2 Faculty of Animal Science and Technology, Yunnan Agricultural University, Kunming 650201, China

**Keywords:** lactating goats, dietary protein levels, milk yield, milk quality, rumen microbiota

## Abstract

Lowering dietary protein content is one of the effective ways to reduce nitrogen (N) emissions and conserve protein feed resources. However, it is unclear how reducing dietary protein levels affects milk quality and the efficiency of N utilization in lactating goats. It is therefore difficult to determine exactly how much reduction in dietary protein levels is optimal. The objective of this study was to evaluate the effects of low-protein diets on milk quality, N balance and rumen microbiota in lactating goats. A total of 50 lactating goats were enrolled in a completely randomized design and maintained on either a diet with 15.82% protein level as the control group (CON) or reduced protein levels with 13.85% (R2 group), 11.86% (R4 group), 9.84% (R6 group) and 7.85% (R8 group), respectively. The results showed that the dry matter intake, milk yield, fecal and urinary N excretion and utilization efficiency of N of lactating goats decreased linearly with reduced dietary protein levels. Specifically, the milk yield was reduced by the R8 group (*p* < 0.05). Furthermore, the R8 group reduced the contents of protein, fat and lactose (*p* < 0.05), but R2 and R4 have no influence (*p* > 0.05). The R6 group decreased protein content only at the 4th week. Fecal and urinary N excretion and utilization efficiency of N reduced linearly with decreasing dietary protein levels (*p* < 0.05). The R8 group affected the relative abundance of rumen microbiota including *Christensenellaceae_R-7_group*, *NK4A214_group* and *UCG-005* (*p* < 0.05). In conclusion, lowering dietary protein levels decreased milk quality and N excretion by altering rumen microbiota in goats during lactation. This phenomenon was most pronounced when the dietary protein level was reduced by 8 percentage points. Nevertheless, dietary protein levels should not be reduced by more than 6 percentage points to ensure normal performance of the goat during lactation.

## Simple Summary

This study evaluated the effects of low-protein diets on milk yield, milk quality, nitrogen (N) metabolism and rumen microbiota of goats during lactation. Goats were fed diets with crude protein levels of 15.82%, 13.85%, 11.86%, 9.84% and 7.85%, respectively. The results showed that dry matter intake (DMI), milk yield and milk quality as well as N emission and utilization in lactating goats decreased linearly with lowering dietary protein levels and affected rumen microbiota composition. Furthermore, there was a strong association between rumen microbiota and milk yield, milk quality and N metabolism. However, the effect was determined by the magnitude of the reduction in crude protein levels. Overall, there was a negative effect of an 8-percentage-point reduction in crude protein levels on lactating goats, and it may be modulated by rumen microbiota. Dietary protein levels should not be reduced by more than 6 percentage points. The tolerance of lactating goats to low-protein diets was confirmed in this experiment. These results provided valuable nutritional strategies for diet formulation in lactating goats. The application of low-protein diets in goats is necessary from the perspectives of environmental protection and social benefits.

## 1. Introduction

There is increasing interest in feeding low-protein diets to ruminants as the cost of milk production increases, coupled with stringent regulations on fertilizer N application in many countries and the challenges involved with ammonia emissions [1,2]. In general, dietary protein is an essential macronutrient affecting the growth and metabolism of animals, production performance and N utilization [3]. It is well known that proteins ingested by animals are deposited in milk, muscle and other tissues as N through a series of metabolic reactions, and the excess is excreted from the body [4]. Several studies have shown that lowering dietary protein levels is considered an effective strategy to reduce nitrogen excretion without compromising animal growth and performance [5,6,7]. However, to the best of our knowledge, the effectiveness of low-protein diets was usually influenced by the magnitude of the reduction in dietary protein levels. Lowering dietary protein levels from 17.5% to 15.0% negatively affected DMI in dairy cows but had no significant effect on milk production [8]. Reducing dietary protein levels by 3 percentage points in growing goats reduced the efficiency of N use and affected rumen fermentation, while lowering diets by 1.5 percentage points had no effect [6]. Other experiments found that no decrease in the DMI of goats was observed when dietary protein levels were reduced from 10.8% to 5.5% [9], 14.0% to 8.0% [10] and 24.7% to 15.8% [11], respectively. It seems to imply that there is some adaptation of goats to diets with lower protein levels.

However, diets with severely low protein content could cause energy–nitrogen metabolism disorders in animals, leading to weight loss, excessive abdominal fat deposition, etc. [12,13]. For specific growth stages of animals, such as lactation, dietary protein levels have received focused attention as an important factor affecting milk production and milk quality [1,8]. Appropriate dietary protein levels are required to ensure optimal lactation performance while meeting the animal’s growth requirements. Dairy goats fed diets with protein levels reduced from 16.0% to 12.2% did not show significant changes in milk yield and lactation quality [14]. Furthermore, previous studies have indicated that changes in the lactation performance of dairy goats were correlated with microbial composition and structure and that ingested dietary protein was an important N source for microorganisms [6,15]. It is generally accepted that microorganisms affect the lactation performance of animals under conditions of adequate dietary protein by participating in the processes of carbohydrate catabolism and protein metabolism [16,17]. However, it is inconclusive whether the effects of low-protein diets on the lactation performance of goats are mediated by microorganisms.

Therefore, considering the scarcity of published data on the application of low-protein diets to lactating goats and the lack of information on the association between low-protein diets and rumen microbiota, we designed the present study and hypothesized that lactating goats could be fed low-protein diets without affecting milk yield and milk quality. However, the magnitude of the reduction in dietary protein levels needs to be controlled, as very low protein levels impair milk yield and milk quality in lactating goats, and this effect may be related to the rumen microbial changes. To verify the hypothesis, this study was to determine the effects of reducing dietary crude protein levels on milk yield, milk quality, N metabolism and microbiota structure in lactating goats. In this way, the appropriate dietary protein level for lactating goats was clarified.

## 2. Materials and Methods

### 2.1. Animal, Experimental Design and Management

**Figure 1:**
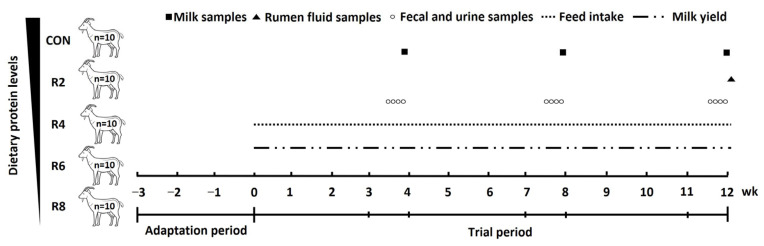
Schematic diagram of the feeding experiment and sample collection. Diets with different levels of protein: CON = 15.82% of protein level; R2 = 13.85% of protein level; R4 = 11.86% of protein level; R6 = 9.84% of protein level; R8 = 7.85% of protein level.

In this study, fifty Yunshang black goats in early lactation (56.22 ± 2.01 DIM) with parity of 2.46 ± 0.17, milk yield of 1.45 ± 0.08 kg/d and BW of 64.85 ± 2.89 kg were used in a completely randomized design. All lactating goats were randomly assigned to one of the five groups, with 10 replicates per group. The lactating goats were fed one of the five diets: a control diet (CON, protein level, 15.82%) and four diets with 13.85% (R2), 11.84% (R4), 9.84% (R6) and 7.85% (R8) inclusion of crude protein, respectively. The ratio of rumen-degradable protein to rumen-undegradable protein was consistent across diets with different protein levels. The control diet was formulated to meet the protein requirement of lactating dairy goats according to the Nutrient Requirements of Small Ruminants 2007 [18]. After a 3-week adaptation period, this was followed by a trial period of 12 weeks (consisting of three periods of 1–4, 5–8 and 9–12 weeks), and the experimental procedure was shown in Figure 1. The nutrient composition of the experimental diets fed as total mixed ration (TMR) is shown in Table 1. During the whole trial period, the lactating goats were fed the experimental diets ad libitum, twice a day with the diet of the corresponding treatment group (0800 and 1700 h) and housed in individual pens (1.7 × 1.3 m) with free access to water. Additionally, the lactating goats were milked twice daily (0700 and 1800 h) using a mechanical milking machine with a controlled vacuum level of 40 kPa and a pulse rate of 100 beats/min, while milk yield was recorded. The mammary glands are pre-soaked and dried before milking. In weeks 4, 8 and 12 of the trial periods, the lactating goats were moved to metabolic cages (1.5 × 1.0 × 1.0 m) with feeders and automatic water collectors for separate collection of total feces and urine. The N balance experiment was carried out to 4 consecutive days after a 3-day environmental adaptation period. During that time, DMI and milk production were recorded daily. At the end of the digestive metabolism experiment, the goats were returned to their original pen.

### 2.2. Dry Matter Intake and Milk Yield

During the 12-week period, the dry matter intake (DMI) of each pen of goats was recorded daily and used to calculate the average weekly DMI (i.e., dynamic DMI). Moreover, DMI was similarly calculated for each experimental phase. From the daily milk yield of goats, weekly and three periods (consisting of three periods of 1–4, 5–8 and 9–12 weeks) milk yield were calculated to monitor the dynamics of milk yield during the experimental period as well as to calculate the fat and protein corrected milk (FPCM) [19].

### 2.3. N Balance and Sample Collection

During the last four days of weeks 4, 8 and 12, all lactating goats were subjected to 4 consecutive days of fecal and urine collection where all feces were collected and recorded daily. Samples were collected and pre-treated according to the method of Lascano and Heinrichs [20] and Yang, et al. [21]. In brief, the feces of the goats were collected daily, and the total fecal weight was recorded; the uncontaminated (including no urine and feed contamination) feces were refrigerated at 4 °C. To prevent the loss of N, an additional H_2_SO_4_ (0.102 mol/L) was added to the above samples at 10% of the sample weight and mixed well before refrigeration. Following the end of the collection period, the 4-day fecal samples from each goat were pooled and mixed well, and a 200 g subsample was taken and dried in a forced-air oven at 65 °C. The samples were then ground through a 0.45 mm sieve and stored at −20 °C for further analysis. For urine samples, urine was collected into glass jars and emptied daily. To prevent ammonia loss, 10% of H_2_SO_4_ (0.102 mol/L) was placed in each urine collection jar at the beginning of the daily urine collection. The daily urine volume was measured, and a 10% aliquot was taken and mixed well with the previous aliquot from each goat. The urine samples were frozen (−20 °C) at the end of each collection period. Finally, urine sub-samples of 50 mL were collected and stored at −20 °C. Two milk samples of 30 mL (one milked in the morning and one milked in the afternoon) were collected at weeks 4, 8 and 12, respectively, a preservative was added and the samples were stored at 4 °C for analysis of milk composition. In the morning of the first day after trial (before morning feeding), rumen fluid was collected from each goat using a rumen catheter into a 5 mL sterile freezing tube and stored at −80 °C for analysis of microbiota structure [22].

### 2.4. Laboratory Analysis

#### 2.4.1. N Metabolism

Samples collected from feed, feces, urine and milk were used to analyze N content. The content of N was analyzed according to the Kjeldahl technique of Association of Analytical Communities [23]. The N intake and N excretion were calculated based on the N content of feed, feces, urine and milk, respectively.

#### 2.4.2. Milk Component

The milk was milked twice a day (0800 and 1600), milk yield was recorded and milk samples were collected in 5% (mL/mL) of the volume of each milk yield. The two milk samples collected were mixed into one sample (half in the morning and half in the afternoon) and stored at 4 °C for further analysis by adding 2-bromo-2-nitropropan-1,3-diol as preservative. The milk samples were analyzed for milk protein, lactose and milk fat using the Milkoscan 6000 instrument (Hillel Rodfors Electric, Hilloerod, Denmark).

#### 2.4.3. Microbiota Structure

Microbial DNA was extracted and sequenced with reference to previous study [24]. The genomic DNA was extracted from the rumen contents using the TIANamp Stool DNA kit (TIANGEN BIOTHEN (BEIJING) Co., Ltd., Beijing, China) following the manufacturer’s instructions. The concentration of total DNA was determined using a Nanodrop 2000 spectrophotometer (Thermo Fisher Scientific, Waltham, MA, USA). The purity of DNA was measured on a 1% agarose gel. The isolated and quality-qualified DNA was used for 16S rRNA gene sequencing. Briefly, primers 341F (5′-CCTAYGGGRBGCASCAG-3′) and 806R (5′-GGACTACNNGGGTATCTAAT-3′) were used to amplify the V3-V4 region of 16S rRNA gene for library construction. PCR conditions were as follows: denaturation at 95 °C for 5 min, followed by denaturation at 95 °C for 45 s, annealing at 55 °C for 50 s, extension at 72 °C for 45 s and finally extension at 72 °C for 10 min for 28 cycles. All PCR products were purified by agarose gel electrophoresis and SanPrep DNA Gel Extraction Kit (Shanghai Sangon Biotechnology Co., Ltd., Shanghai, China). The libraries were sequenced on the Illumina HiSeq platform according to the methods and processes of the Majorbio Bio-Pharm Technology Co., Ltd., (Shanghai, China) after being tested and characterized for quality. All reads have been deposited in the National Center for Biotechnology Information (NCBI, Bethesda, MD, USA) and are available in the Short Read Archive (SRA) under the accession number PRJNA1142682. The raw sequencing data were processed using the Quantitative Insights into Microbial Ecology (QIIME2) pipeline [25]. Sequences were grouped into the same operational taxonomic unit (OTU) at a similarity threshold of 97%. Alpha diversity indices (Shannon, Chao1, Simpson, and ACE) were analyzed using Mothur software (v1.30.2). Bray–Curtis distance-based principal component analysis (PCA) was performed using the R language (v 3.3.1).

### 2.5. Calculations and Statistical Analysis

Given that intake and excretion of N have been calculated, the N balance was further evaluated by the following indicators [6]:

N retention = N intake − (Fecal N excretion + Urinary N excretion)

Apparent digestibility of N = (N intake − Fecal N excretion)/N intake × 100%

Utilization efficiency of N = N retention/N intake × 100%

FPCM was calculated using the method by Battelli et al. [20], according to the following formula:

FPCM = milk yield × (0.26 + 0.1352 × Fat% + 0.079 × Protein%)

The normality of the distribution was evaluated using the Shapiro–Wilk test before data analysis [26]. Experimental data (excluding 16S rRNA sequencing data) with the pen as a statistical unit were analyzed using PROC MIXED of SAS 9.4 (SAS Inst. Inc., Cary, NC, USA). Firstly, linear, quadratic and cubic effects of reducing dietary protein levels were assessed by orthogonal polynomial contrasts. Moreover, to observe the effects of dietary treatments, data were subjected to two-way ANOVA analysis using the PROC MIXED of SAS. Statistical model included the effects of the feeding time period (T), dietary protein levels (R) and T × R. The following linear model with repeated correlation structure was considered:

Y_ij_ = m + T_i_ + R_j_ + (T_i_ × R_j_) + e_ij_

where Y_ij_ was the response variable; m was the overall mean; T_i_ was the fixed effect of the feeding time period (i = 1 to 3); R_j_ was the dietary protein levels (j = 1 to 5); T_i_ × R_j_ was the interaction of feeding time period and dietary protein levels; e_ij_ is the random error. For data on weekly DMI and milk yield, the differences between the R2, R4, R6 and R8 groups and the control group were analyzed using two-tailed Student’s *t*-test, respectively. The microbial relative abundance data from 16S rRNA sequencing were analyzed using Kruskal–Wallis one-way ANOVA. Spearman’s rank correlation coefficients were used to assess the correlation between microorganisms and environmental factors including milk quality and N utilization. Statistical significance of all data was considered at *p* < 0.05, with *p* ≥ 0.05 to *p* < 0.10 representing a trend. However, given that this experimental design was constrained by practical limitations, including ethical guidelines for animal use, logistical challenges in maintaining large cohorts under controlled conditions and resource availability, more sample sizes are needed to ensure statistical power. Further experiments will prioritize large sample sizes.

## 3. Results

### 3.1. Dry Matter Intake, Milk Yield and Feed Efficiency

As shown in Table 2, there was a linear decrease in the DMI, milk yield and FPCM of lactating goats with reducing dietary protein levels during the whole experimental period (*p* < 0.01). Similar effects were also found in periods 1 to 4 weeks, 5 to 8 weeks and 9 to 12 weeks, respectively (*p* < 0.01). In addition, lowering dietary protein levels quadratically affected DMI (*p* < 0.01), milk yield (*p* = 0.01) and FPCM (*p* = 0.02), with minimal values at the R8 group. There was a significant effect of feeding time period for DMI, milk yield and FPCM (*p* < 0.01). There was a trend interaction between feeding time and protein level for DMI (*p* = 0.08). However, no significant effect was observed on feed efficiency, including milk yield/DMI and FPCM/DMI, of lactating goats fed low-protein diets (*p* > 0.05).

**Figure 2:**
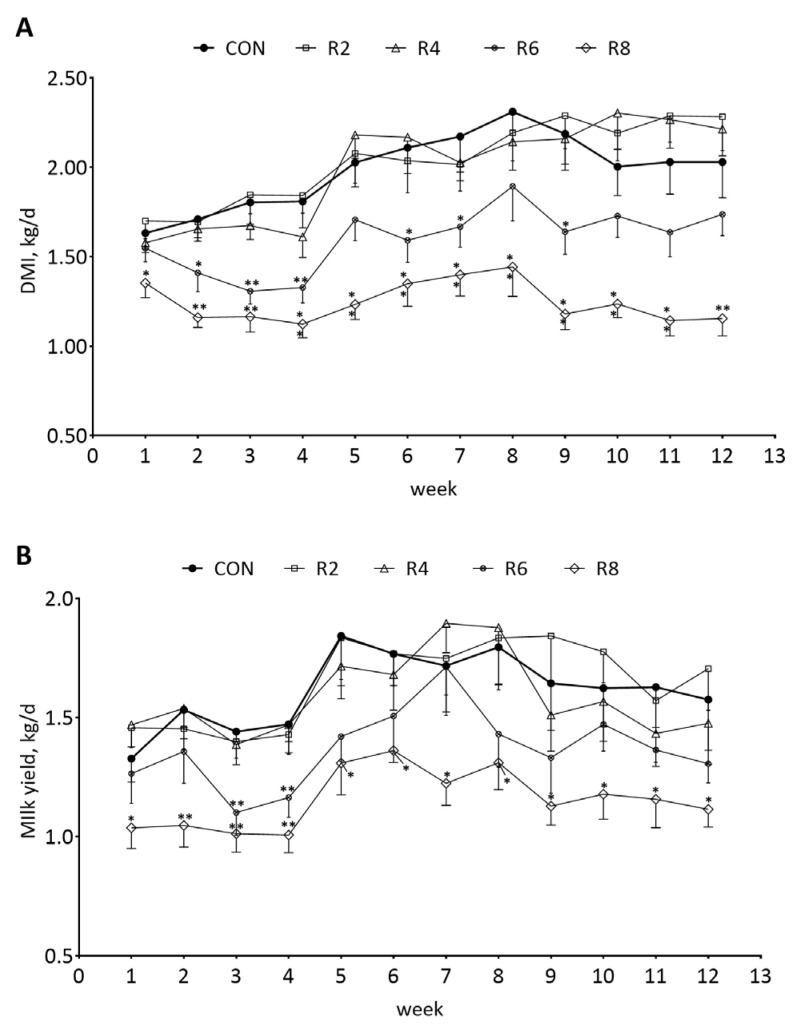
Effects of dietary protein levels on the weekly DMI (**A**) and milk yield (**B**) of lactating goats. Diets with different levels of protein: CON = 15.82% of protein level; R2 = 13.85% of protein level; R4 = 11.86% of protein level; R6 = 9.84% of protein level; R8 = 7.85% of protein level. * Indicates a significant difference (*p* < 0.05) compared to the CON group using the R2, R4, R6 and R8 groups, respectively. ** indicates a highly significant difference (*p* < 0.01).

To investigate the changes in DMI and milk yield in lactating goats fed low-protein diets, we analyzed the dynamics of DMI and milk yield weekly. The weekly DMI (Figure 2A) and milk yield (Figure 2B) of lactating goats were significantly decreased when the R8 group compared with the CON group during the experimental period (*p* < 0.05). There were similar effects on DMI and milk yield in the R6 group in the 4th week (first phase, 1 to 4 weeks) only (*p* < 0.05), while the R2 and R4 groups had no significant effects on weekly DMI (Figure 2A) and milk yield (Figure 2B) of lactating goats compared with the CON group (*p* > 0.05).

### 3.2. Milk Composition and Content

As presented in Table 3, for the milk composition (%), a linear decrease in milk protein was observed in lactating goats with reducing dietary protein levels (*p* < 0.01), and a quadratic effect was observed in the period of 1 to 4 weeks (*p* = 0.03) with minimal values at the R8 group. However, there was no effect of dietary protein levels for fat and lactose in milk (*p* > 0.05). The feeding time period or feeding time period by dietary protein levels interaction had no significant effects on protein, fat and lactose during the whole experimental phase (*p* > 0.05).

Regarding the milk content (g/d), the protein, fat and lactose were linearly reduced with decreasing dietary protein levels during the whole experimental phase (*p* < 0.01) and the periods of 1 to 4 weeks (*p* < 0.01), 5 to 8 weeks (*p* < 0.05) and 9 to 12 weeks (*p* < 0.01). Lowering dietary protein levels quadratically affected the contents of protein (*p* = 0.01), fat (*p* = 0.04) and lactose (*p* = 0.03), respectively. In particular, protein, fat and lactose contents were lowest in the R8 group. In contrast, the milk fat–protein ratio within the whole experimental phase (*p* < 0.01) and the periods of 1 to 4 weeks (*p* = 0.02) and 5 to 8 weeks (*p* = 0.02) increased linearly with decreasing dietary protein levels, presenting the highest ratio in the R8 group. There was no effect of feeding time period by dietary protein levels interaction for milk content (*p* > 0.05).

### 3.3. N Balance

As shown in Table 4, there was a linear decrease in N intake (*p* < 0.01) and fecal N excretion (*p* < 0.01) during the whole experimental period, as dietary protein levels reduced, and the same changes were observed in urinary N excretion (*p* = 0.02) and milk N excretion (*p* < 0.01), which were all lower in the R8 group. For the different feeding stages, both N intake and fecal N excretion showed linear decreases (*p* < 0.01), while similar influences were observed for urinary N excretion (*p* = 0.03) and milk N excretion (*p* = 0.01) from 1 to 4 weeks. There was no significant interaction effect between feeding time period and dietary protein levels for N intake, fecal N excretion, urinary N excretion and milk N excretion (*p* > 0.05). The N retention was linearly reduced as the dietary protein levels decreased (*p* < 0.01), but the apparent digestibility of N was linearly increased in the stage of 1 to 4 weeks (*p* < 0.05). The utilization efficiency of N was significant affected by feeding time period, as evidenced by higher values measured at 9–12 weeks (*p* = 0.04). The utilization efficiency of N was affected by dietary protein level, with the largest reduction in the R8 group (*p* < 0.01).

### 3.4. Bacterial Richness and Diversity

**Figure 3:**
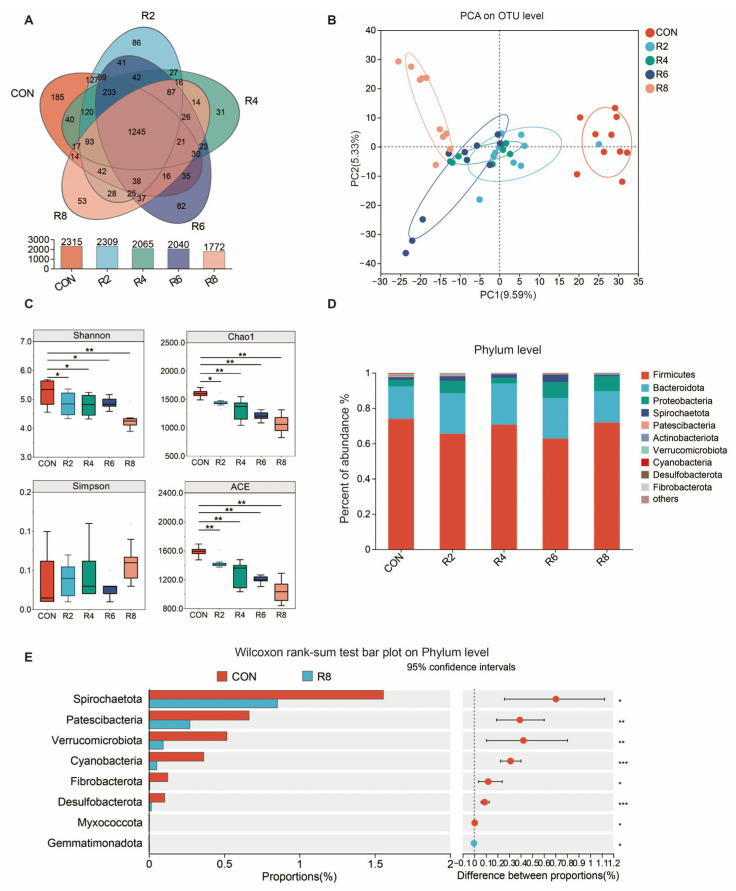
Effect of dietary protein level on alpha diversity and phylum-level flora differences in the rumen microbiota of lactating goats. Diets with different levels of protein: CON = 15.82% of protein level; R8 = 7.85% of protein level. (**A**) A Venn plot for identifying the number of species based on the level of OUTs. (**B**) Principal component analysis ordination plots of OTUs based on the Bray–Curtis distance metric. (**C**) Changes in alpha diversity indices Shannon, Chao1, Simpson and ACE. (**D**) Relative abundance of rumen microbiota at the phylum level. (**E**) Plot of the R8 group compared to the CON group based on Wilcoxon rank-sum test at the genus level. *, **, and *** represent *p* < 0.05, *p* < 0.01, and *p* < 0.001, respectively.

To investigate whether the effects of low-protein diets on milk quality and N metabolism were correlated with altered microbiota structure, we examined the composition of the microbiota in the rumen. A total of 1245 OUTs were identified in the rumen of lactating goats, and 185, 86, 31, 82 and 53 specific OTUs were obtained from six groups with CON, R2, R4, R6 and R8, respectively (Figure 3A). PCA analysis showed that the low-protein diets resulted in a significant separation of the microbial community from the CON group, with the highest significance in the R8 group (Figure 3B). The Shannon, Chao1 and ACE indices decreased in groups R2, R4, R6 and R8 compared to the CON group, with the lowest index in group R8 (Figure 3C) (*p* < 0.05).

### 3.5. Ruminal Microbial Differences at the Phylum and Genus Level

**Figure A1:**
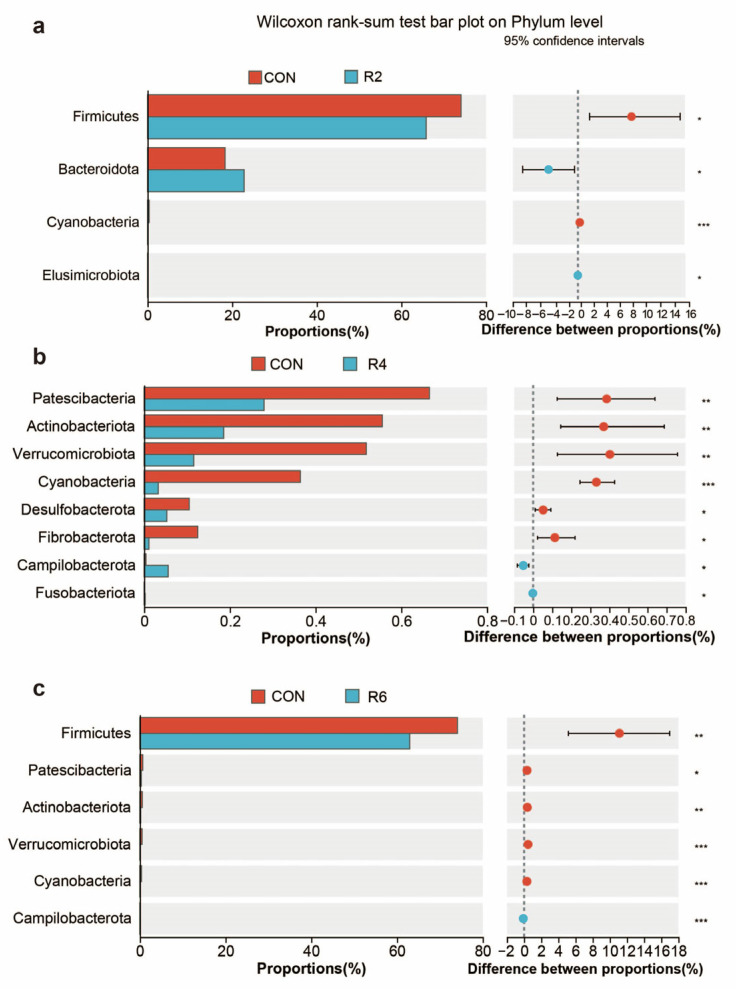
Effects of dietary protein levels on the rumen microorganisms at phylum level in lactating goats. Diets with different levels of protein: CON = 15.82% of protein level; R2 = 13.85% of protein level; R4 = 11.86% of protein level; R6 = 9.84% of protein level; R8 = 7.85% of protein level. (**a**) Plot of the R2 group compared to the CON group based on Wilcoxon rank-sum test at the genus level. (**b**) Plot of the R4 group compared to the CON group based on Wilcoxon rank-sum test at the genus level. (**c**) Plot of the R6 group compared to the CON group based on Wilcoxon rank-sum test at the genus level. *, **, and *** represent *p* < 0.05, *p* < 0.01, and *p* < 0.001, respectively.

At present in Figure 3, the dominant bacteria in the rumen were Firmicutes, Bacteroidota, Proteobacteria, Spirochaetota, Patescibacteria and Actinobacteriota (Figure 3D) and were partially affected by the low-protein diets. The R8 group significantly reduced the relative abundance of Spirochaetota, Patescibacteria, Verrucomicrobiota, Cyanobacteria, Fibrobacterota, Desulfobacterota, Myxococcota and Gemmatimonadota when compared with the CON group (Figure 3E) (*p* < 0.05). There were similar influences in the R2 (Figure A1a), R4 (Figure A1b) and R6 (Figure A1c) groups when compared to the CON group, but the largest effects were observed in group R8 overall.

**Figure 4:**
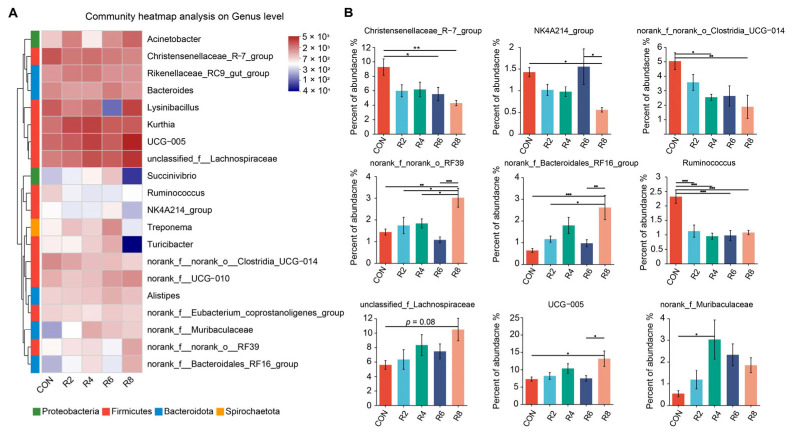
Effects of dietary protein levels on the rumen microbiota at genus level in lactating goats. Diets with different levels of protein: CON = 15.82% of protein level; R2 = 13.85% of protein level; R4 = 11.86% of protein level; R6 = 9.84% of protein level; R8 = 7.85% of protein level. (**A**) The Top 20 microorganisms in relative abundance at the genus level in the rumen. (**B**) Significant differences between treatments were determined using the Kruskal–Wallis test. *, **, and *** represent *p* < 0.05, *p* < 0.01, and *p* < 0.001, respectively.

**Figure A2:**
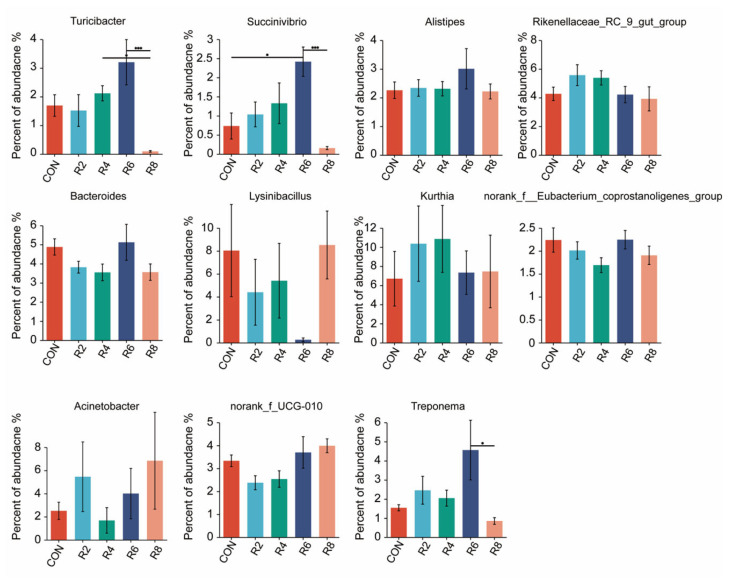
Effects of dietary protein levels on the rumen microorganisms at genus level in lactating goats. Diets with different levels of protein: CON = 15.82% of protein level; R2 = 13.85% of protein level; R4 = 11.86% of protein level; R6 = 9.84% of protein level; R8 = 7.85% of protein level. * and *** represent *p* < 0.05 and *p* < 0.001, respectively.

At the genus level, we further analyzed microorganisms with top 20 relative abundance and observed that lowering dietary protein levels resulted in some differences in rumen microbial profiles (Figure 4A). Specifically, when compared with the CON group, the R8 group significantly decreased the relative abundance of *Christensenellaceae_R-7_group*, *NK4A214_group*, *norank_f_norank_o_Clostridia_UCG-014* and *Ruminococcus*, while increasing the relative abundance of *norank_f_norank_o_RF39*, *unclassified_f_Lachnospiraceae*, *norank_f_Bacteroidales_RF16_ group* and *UCG-005* (Figure 4B) (*p* < 0.05). The relative abundance of *Christensenellaceae_R-7_group* and *Ruminococcus* was decreased by the R6 group, but *Succinivibrio* was increased (Figure A2) (*p* < 0.05). The reduction in the relative abundance of *norank_f_norank_o_Clostridia_UCG-014* and *Ruminococcus* was observed in the R4 group compared to the CON group (*p* < 0.05), whereas *norank_f_Muribaculaceae* was elevated (*p* < 0.05), and no significant effect was observed for other microorganisms (Figure 4B and Figure A2) (*p* > 0.05). However, there were no significant influences in the top 20 microorganisms (except for *Ruminococcus*) between the R2 and CON groups (Figure 4B and Figure A2) (*p* > 0.05).

### 3.6. Correlation

**Figure 5:**
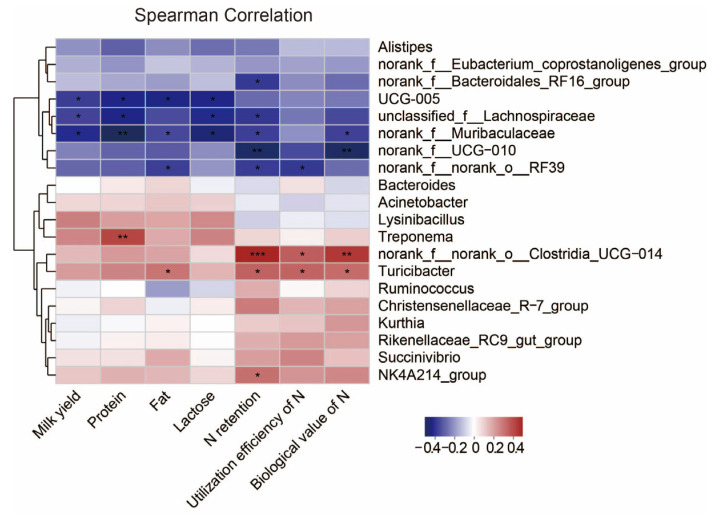
Correlation of rumen microbiota in dairy goats with milk yield, milk quality and N metabolism. Heatmaps based on Spearman correlation coefficients showing the relationship between the top 20 rumen microorganisms at genus level and milk yield, milk quality and N metabolism. *, **, and *** represent *p* < 0.05, *p* < 0.01, and *p* < 0.001, respectively.

Next, we investigated associations between the rumen microbiota and milk yield, milk quality and N metabolism. We calculated Spearman correlation coefficients and visualized them using a heatmap (Figure 5). The milk yield and the concentrations of protein and lactose in milk were both negatively correlated with *unclassified_f_Lachnospiraceae*, *norank_f_Muribaculaceae* and *UCG-005* (*p* < 0.05). The milk protein concentration was positively correlated with *Treponema* (*p* < 0.05). The milk fat concentration was positively correlated with *Turicibacter*, while negatively correlated with *UCG-005*, *norank_f_Muribaculaceae* and *norank_f_norank_o_RF39* (*p* < 0.05). N utilization was negatively correlated with *norank_f_Bacteroidales_RF16_group*, *unclassified_f_Lachnospiraceae*, *norank_f_Muribaculaceae*, *norank_f_ UCG-010* and *norank_f_norank_o_RF39*, whereas *norank_f_norank_o_Clostridia_UCG-014*, *Turicibacter* and *NK4214_group* were positively correlated with it (*p* < 0.05).

## 4. Discussion

The appropriate level of reduction in dietary protein levels remains a concern, although low-protein diets are widely accepted and used in animal production. Thus, we hypothesized that lactating goats could be fed low-protein diets without compromising performance, but the magnitude of the reduction in dietary protein levels would need to be controlled. Notably, our results confirmed the above hypothesis. Reducing protein levels in dairy cow diets to about 140–150 g/kg diet DM had no effects on performance, health, or fertility in previous study [2]. Nevertheless, decreasing the dietary protein concentration from 173 g/kg DM to 144 g/kg DM decreased DM intake and milk quality in dairy cows [27,28]. The effects of lowering diet protein levels on feed intake are still inconsistent for ruminants. Feeding a low-protein diet with a 3.7% and 3.1% reduction in protein level during peak and late lactation, respectively, reduced feed intake in dairy cows [29]. In the present study, a linear decrease in the DMI of lactating goats was observed with decreasing dietary protein levels and was remarkably and negatively affected especially when the protein level was reduced by 8 percentage points as compared to the control diet. However, considering the observed values of dynamic DMI, a 2- and 4-percentage-points reduction in dietary protein levels had no significant effect on DMI, respectively. Our results support the concept of previous studies that goats are more tolerant when faced with nutritional changes in their diets [30]. Insufficient dietary protein intake can lead to a sub-healthy state of the animal and metabolic disorders in the body thus affecting growth [3,12,31]. In particular, lactating goats have a greater need for dietary protein, which may disrupt metabolism if not consumed adequately [32]. It has also been suggested that low-protein diets lead to a reduction in the availability of effective N in the rumen, which inhibits the activity of fiber-degrading bacteria and ultimately leads to lower feed intake [2]. Therefore, the consumption of diets with excessively low protein levels by lactating goats has the potential effect of reducing production performance including growth and lactation efficiency. Currently, lowering dietary protein levels have been frequently reported to decrease lactation performance, but the appropriate low protein level for lactating animals has not been conclusively determined [2]. For example, Giallongo et al. [33] found that reducing dietary protein level from 17.0% to 15.0% in dairy cows decreased milk yield by approximately 4.3 kg/d. According to Arieli et al. [34], the milk yield of dairy goats in each period was unaffected by low-protein diet. In our study, lowering dietary protein levels linearly reduced milk yield and FPCM, and this phenomenon was due to a greater extent to the reduction in protein levels up to 8 percentage points. Specifically, reducing dietary protein levels by 2 and 4 percentage points had no significant effect on weekly milk yield, whereas a 6-percentage-point reduction had a significant reduction effect only in the first four weeks, which disappeared over time. Hence, it seems to imply that dietary quality and metabolizable protein supply of this experimental diet were sufficient to meet the lactation requirements of lactating goats even if the dietary protein level was reduced by 6 percentage points. Furthermore, dietary protein is vital for animal growth and health, and it is an important source of nutrients involved in milk synthesis. To our knowledge, few studies have evaluated the influence of reduced dietary protein levels on milk quality in dairy goats. In the current study, regarding the protein content in milk, a significant negative effect was induced by the reduction in dietary protein levels at each period, while there was no effect on fat and lactose content. As mentioned in previous studies, the conversion process between protein, fat and lactose in the milk of lactating animals was extremely complex, and dietary protein was the primary factor affecting the protein content of milk. Milk quality in lactating goats is determined by protein, fat and lactose content, and they are significantly and negatively affected by lower dietary protein levels. This effect may be influenced by the dual effect of dietary protein levels on lactation and N metabolism.

Efficiency of N utilization by animals is one of the main factors determining the amount of N deposited in body tissues and animal products [1]. Increasing evidence supports the view that the first metabolic pathway affected in animals is N metabolism when dietary protein concentration is reduced [35]. It is generally accepted that a reduction in N intake decreases N excretion in feces and urine [5,20]. This phenomenon is found in the reduction in dietary protein levels from 13% to 10% in growing goats and from 16.5% to 12% in late-lactation cows [6,36]. In our study, a linear decrease in N intake in lactating goats was observed with decreasing dietary protein levels throughout the experimental period, and this effect was particularly enhanced after reducing protein levels by 8 percentage points compared to the control group. We further found that the N excretion from the feces of dairy goats decreased linearly with decreasing dietary protein levels. Therefore, N intake is the primary driver determining N excretion. However, there was no significant effect on fecal and urinary N excretion in goat kids fed diets with 10%, 13% and 16% crude protein levels, respectively [37]. This may be attributed to the fact that N utilization in animals is influenced by differences in physiological and metabolic functions at different growth stages. In particular, for lactating goats or cows, the ingested N is excreted in the milk in addition to feces and urine [7]. Our experimental results demonstrated that milk N excretion decreases with decreasing dietary protein levels. Notably, our results found that the percentage reduction in fecal N excretion in lactating goats fed low-protein diets exceeded that of urinary and milk N excretion, respectively. The results were essentially consistent with previous studies [6]. In addition, we found that lowering dietary protein levels resulted in a linear decrease in N retention. This may be one of the major reasons for the change in protein content in milk. Similar results have been found in previous experiments where goats were fed low-protein diets of 17.6%, 11.7% and 8.7% with N retention of 4.45, 3.22 and 1.56 g/day, respectively [38]. In fact, there was an intimate relationship between the retention and utilization of N. The apparent digestibility of N in this study increased linearly as dietary protein levels decreased, while the utilization efficiency of N decreased. It is probably explained by the fact that the apparent digestibility of N in the whole gut can be affected by several factors including endogenous N loss and re-circulation of N through the rumen–hepatic pathway. Notably, N utilization showed a significant decrease after 6 and 8 percentage points of reduction in protein level, respectively, whereas a 4-percentage-point decrease had no significant effect and was even numerically greater than that of the control group. Overall, there was a negative influence on both N deposition and utilization when the dietary protein level was reduced by 6 or 8 percentage points. Hence, a 4-percentage-point reduction in dietary protein was an effective strategy to reduce N emissions without affecting N utilization from the viewpoint of N metabolism in lactating goats.

Ruminant urea-N produced in the liver is continually recycled, thereby supplying N to the rumen microbial community [39]. Nevertheless, this mechanism can be compromised and lead to an alteration of microbial structure and composition when dietary N supply is limited [32]. The Chao1 and ACE indices were used to evaluate microbial abundance, with their elevated values representing a higher number of species [40]. Both Simpson and Shannon indexes were used to assess microbial diversity. Among them, a higher Shannon index represents higher community diversity, whereas the opposite is true for the Simpson index. Reduced microbial diversity was found to lead to a reduction in short-chain fatty acids in a model of inflammatory gastrointestinal disease [41]. In our study, we found that the Chao1, ACE and Shannon decreased significantly with reducing dietary protein levels. Feeding a low-protein diet (especially with an 8-percentage-point reduction in protein level) reduced the number of OTUs of rumen microbiota in lactating goats in our experiment, supporting the findings of Zhang et al. [6], who also reported similar results when dietary protein levels were reduced from 13% to 10%. It was one of the reasons why the low-protein diets reduced the Chao1 and ACE indices of rumen microbiota in the present experiment. The dominant bacteria in the rumen of goats were the Firmicutes, Bacteroidetess and Proteobacteria in the previous studies [6,42]. This finding was similarly verified by our experiment. The abundance of microorganisms at the phylum level was affected increasingly as dietary protein levels were reduced, as evidenced by the fact that reducing protein levels by 8 percentage points resulted in a significant reduction in Spiochaetota, Patescibacteeria, Verrucmicrobiota, Cyanobacteria, Fibrobacterota and Desulfobacterota. The Spirochetes mainly hydrolyzed complex polysaccharides in plant cell walls, degraded proteins and produced B vitamins in the rumen [43]. The Cyanobacteria contribute to the increase of milk yield in lactating cows [17]. Moreover, Fibrobacterota and Desulfobacterota phylum are potential factors affecting milk yield, milk protein and milk fat in dairy cows [31,44]. The relative abundance of phylum level microorganisms decreased with the R8 diet in this experiment, which could explain the reduction in milk yield and protein content. To further investigate the effects of microbes on milk quality and N metabolism in lactating goats exposed to different dietary protein levels, we analyzed the variation of microorganisms at the genus level. Several microorganisms showed significant changes with decreasing protein levels in this study. For example, the relative abundances of *norank_f_norank_o_Clostridia_UCG-014* and *Ruminococcus* were lower in the R4 diet compared to the control, and similarly lower for *Christensenellanceae_R-7_group* and *Ruminococcus* in the R6 diet. It is noteworthy that, fundamentally consistent with the effects of dietary protein levels on milk yield, milk protein and N utilization efficiency, there were more pronounced microbial effects in lactating goats fed R8 diets. This confirmed the previous view that protein-deficient diets limited microbial activity in the rumen and impaired digestion [32]. Specifically, the relative abundances of *Christensenellaceae_R-7_group*, *NK4A214_group*, *norank_f_norank_o_Clostridia*, *_UCG-014* and *Ruminococcus* were decreased, and the relative abundances of *norank_f_norank_o_RF39*, *norank_f_Bacteroidales_RF16_group* and *UCG-005* were increased when R8 group is compared with CON group. Previous studies indicated that *Christensenellanceae_R-7_group* is associated with catabolism of proteins and utilization of N from feeds [45] and improves gastrointestinal health in dairy goats, promoting protein degradation and nutrient digestion and absorption [46]. Additionally, *Christensenellanceae_R-7_group* belongs to fiber-degrading bacteria. A decrease in *Christensenellanceae_R-7_group* leads to a lower rate of fiber degradation, increasing the animal’s satiety and thus reducing feed intake [47]. The above possibilities were supported by the fact that in the present study, the DMI and N utilization efficiency of lactating goats in the R8 group was lower than that of the CON group. The genus *Ruminococcus* is responsible for breaking down fibrous plant matter to produce short-chain fatty acids such as acetate, formate and succinate, which further affects the milk yield [16]. It appears that these potential effects of microorganisms could be used to explain the changes in milk yield and N metabolism resulting from low protein in this experiment. Previous study pointed out that milk yield was directly or indirectly affected by rumen microorganisms [48]. To evaluate the possibility of this hypothesis, we performed a correlation analysis between microorganisms at genus level top 20 and milk yield, milk protein, milk fat and N utilization efficiency, etc., respectively. It was found that both *unclassified_f_Lachnospiraceae*, *UCG-005* and *norank_f_Muribaculaceae* were significantly negatively correlated with milk yield and milk protein. *Christensenellaceae_R-7_group* was significantly positively correlated with N retention and utilization efficiency of N, while *norank_f_norank_o_RF39* was negatively correlated with N retention and utilization efficiency of N. These associations have been found in previous studies. Jiang et al. [49] reported that relationships between the relative abundance of *UCG-005* and trends in milk yield and milk protein were generally consistent under conditions where cows received gluten-wheat bran mixture treatments. The influence of *Christensenellanceae_R-7_group* and *norank_f_Muribaculaceae* on N utilization and milk production performance in animals has also been demonstrated in previous studies [46]. Overall, rumen microbes may be one of the factors affecting milk yield, milk quality and nitrogen metabolism in lactating goats.

## 5. Conclusions

Reducing dietary crude protein levels by 2 and 4 percentage points, respectively, has no effect on the production performance of lactating goats. A low-protein diet with a 6-percentage-point reduction only resulted in lower milk protein in phase 1 to 4 weeks and lower N retention. Once the dietary protein level was reduced by 8 percentage points, a decrease in milk yield, FPCM, milk quality and N utilization efficiency was observed. Furthermore, there was a significant link between these changes and the structure and composition of the rumen microbiota. It implies that it seems possible for lactating goats to tolerate lower dietary protein by employing microbial interventions. Overall, diets should not be reduced by more than 6 percentage points of protein level to ensure normal performance of goats in lactation. More research is needed to further substantiate these inferences.

## Figures and Tables

**Table 1:** Ingredients and chemical of diets fed to lactating goats (DM basis).

Items	Diets ^1^				
	CON	R2	R4	R6	R8
Ingredients (%)					
Corn silage	40.46	41.33	41.21	42.14	40.14
Rice straw	9.54	8.67	8.79	7.86	9.86
Corn	22.35	26.68	31.28	35.48	38.84
Corn starch	2.81	2.54	2.15	1.45	1.21
Wheat bran	1.03	1.43	1.68	1.92	2.00
Soybean meal	16.35	12.55	8.98	5.91	3.25
Rapeseed meal	2.61	2.34	1.75	1.54	1.22
Soybean oil	1.65	1.24	0.94	0.48	0.12
NaCl	0.50	0.50	0.50	0.50	0.50
Mineral/vitamin premix ^2^	1.00	1.00	1.00	1.00	1.00
Calcium carbonate	1.33	1.33	1.31	1.31	1.45
Monocalcium phosphate	0.37	0.39	0.41	0.41	0.41
Total	100.00	100.00	100.00	100.00	100.00
Nutrient levels (%) ^3^					
ME (MJ/kg)	9.66	9.65	9.65	9.64	9.65
CP ^4^	15.82	13.85	11.86	9.84	7.85
NDF ^5^	34.42	34.58	35.02	35.22	36.46
ADF ^6^	17.20	17.15	16.92	16.87	16.75
Calcium	0.85	0.85	0.84	0.84	0.83
Total phosphate	0.66	0.66	0.64	0.63	0.66
Organic matter	96.43	96.62	96.85	96.90	97.21
Ether extract	4.21	4.18	4.11	3.87	3.62
Ash	3.57	3.38	3.15	3.10	2.79

^1^ Diets with different levels of protein: CON = 15.82% of protein level; R2 = 13.85% of protein level; R4 = 11.86% of protein level; R6 = 9.84% of protein level; R8 = 7.85% of protein level. ^2^ Trace Mineral and Vitamin premix (DM basis): 145 mg/kg Fe, 80 mg/kg Zn, 20 mg/kg Cu, 98 mg/kg Mn, 2.5 mg/kg I, 0.35 mg/kg Se, 0.65 mg/kg Co; 10,000 IU/kg Vitamin A, 1000 IU/kg Vitamin D, 200 IU/kg Vitamin E. ^3^ The nutrient levels were the measured values. ^4^ CP: crude protein. ^5^ NDF: Neutral detergent fiber. ^6^ ADF: Acid detergent fiber.

**Table 2:** Effects of dietary protein levels on the DMI, milk yield and feed efficiency of lactating goats.

Item	Diets ^1^					SEM		*p*-Value ^2^			
	CON	R2	R4	R6	R8		T	T × R	L	Q	C
IBW ^3^ (kg)	64.63	65.31	64.77	64.73	64.83	0.96			0.96	0.86	0.66
DMI ^4^ (kg/d)											
Overall	1.99	2.04	2.00	1.60	1.24	0.09	<0.01	0.08	<0.01	<0.01	0.52
1 to 4 week	1.74	1.77	1.63	1.40	1.20	0.07			<0.01	0.04	0.37
5 to 8 week	2.16	2.08	2.13	1.72	1.36	0.11			<0.01	0.02	0.84
9 to 12 week	2.06	2.26	2.24	1.68	1.18	0.10			<0.01	<0.01	0.37
Milk yield (kg/d)											
Overall	1.61	1.65	1.58	1.37	1.16	0.11	<0.01	0.98	<0.01	0.01	0.61
1 to 4 week	1.44	1.44	1.47	1.23	1.03	0.07			<0.01	0.02	0.98
5 to 8 week	1.78	1.80	1.79	1.52	1.30	0.13			<0.01	0.14	0.85
9 to 12 week	1.62	1.73	1.50	1.37	1.14	0.12			<0.01	0.19	0.51
FPCM ^5^											
Overall	2.10	2.13	2.03	1.78	1.45	0.09	<0.01	0.99	<0.01	0.02	0.91
1 to 4 week	1.86	1.85	1.86	1.57	1.29	0.10			<0.01	0.04	0.98
5 to 8 week	2.28	2.29	2.27	1.95	1.65	0.19			0.01	0.20	0.96
9 to 12 week	2.16	2.24	1.95	1.82	1.41	0.16			<0.01	0.17	0.85
Feed efficiency											
Milk yield/DMI											
Overall	0.86	0.85	0.81	0.87	0.96	0.06	0.30	0.80	0.25	0.17	0.68
1 to 4 week	0.87	0.84	0.90	0.87	0.88	0.07			0.82	0.98	0.75
5 to 8 week	0.86	0.92	0.85	0.91	1.02	0.10			0.36	0.58	0.55
9 to 12 week	0.85	0.81	0.67	0.82	0.99	0.09			0.29	0.04	0.67
FPCM/DMI											
Overall	1.12	1.10	1.03	1.13	1.20	0.08	0.47	0.89	0.47	0.23	0.91
1 to 4 week	1.14	1.08	1.14	1.12	1.11	0.09			0.94	0.99	0.72
5 to 8 week	1.10	1.17	1.08	1.17	1.29	0.14			0.37	0.58	0.67
9 to 12 week	1.13	1.05	0.87	1.09	1.21	0.12			0.58	0.07	0.98

^1^ Diets with different levels of protein: CON = 15.82% of protein level; R2 = 13.85% of protein level; R4 = 11.86% of protein level; R6 = 9.84% of protein level; R8 = 7.85% of protein level. ^2^ *p*-value: T = feeding time period; R = dietary protein levels; L = linear effect of protein levels; Q = quadratic effect of protein levels; C = cubic effect of protein levels. ^3^ IBW = initial average body weight. ^4^ DMI = dry matter intake. ^5^ FPCM = fat and protein corrected milk, i.e., milk yield × (0.26 + 0.1352 × Fat% + 0.079 × Protein%).

**Table 3:** Effects of dietary protein levels on the milk quality of lactating goats.

Item	Diets ^1^					SEM		*p*-Value ^2^			
	CON	R2	R4	R6	R8		T	T × R	L	Q	C
Milk composition											
Protein (%)											
Overall	3.70	3.59	3.60	3.50	3.24	0.07	0.13	0.73	<0.01	0.12	0.21
1 to 4 week	3.69	3.71	3.78	3.54	3.29	0.10			<0.01	0.03	0.84
5 to 8 week	3.77	3.49	3.48	3.50	3.32	0.10			0.01	0.55	0.14
9 to 12 week	3.63	3.56	3.55	3.45	3.13	0.12			0.01	0.20	0.46
Fat (%)											
Overall	5.55	5.49	5.43	5.64	5.50	0.14	0.14	0.91	0.91	0.86	0.42
1 to 4 week	5.47	5.46	5.28	5.49	5.52	0.23			0.85	0.59	1.00
5 to 8 week	5.31	5.38	5.34	5.53	5.58	0.25			0.39	0.85	0.97
9 to 12 week	5.86	5.63	5.67	5.90	5.39	0.23			0.36	0.66	0.16
Lactose (%)											
Overall	4.66	4.65	4.81	4.59	4.64	0.09	0.76	0.57	0.74	0.43	0.72
1 to 4 week	4.53	4.50	5.14	4.54	4.58	0.23			0.83	0.20	0.97
5 to 8 week	4.79	4.72	4.79	4.63	4.66	0.18			0.52	0.96	0.93
9 to 12 week	4.65	4.73	4.51	4.59	4.67	0.18			0.87	0.64	0.59
Milk content											
Protein (g/d)											
Overall	59.42	59.48	56.90	47.90	37.61	2.57	<0.01	0.97	<0.01	0.01	0.87
1 to 4 week	53.22	53.16	55.59	43.15	33.75	3.00			<0.01	<0.01	0.95
5 to 8 week	66.72	63.76	62.44	53.06	43.26	5.11			<0.01	0.26	0.90
9 to 12 week	58.33	61.53	52.66	47.51	35.83	4.37			<0.01	0.12	0.69
Fat (g/d)											
Overall	89.77	90.69	86.16	77.47	64.05	4.47	<0.01	0.99	<0.01	0.04	0.98
1 to 4 week	79.07	78.19	77.33	67.39	56.12	5.01			<0.01	0.12	0.93
5 to 8 week	95.58	97.16	96.62	84.32	71.69	8.84			0.04	0.23	0.95
9 to 12 week	94.67	96.74	84.54	80.69	61.34	7.41			<0.01	0.22	0.96
Lactose (g/d)											
Overall	75.49	76.64	75.99	62.64	53.90	3.83	<0.01	0.66	<0.01	0.03	0.60
1 to 4 week	65.07	63.94	75.28	55.32	47.55	4.28			<0.01	0.01	0.99
5 to 8 week	85.72	84.19	85.57	69.69	60.77	6.53			<0.01	0.20	0.85
9 to 12 week	75.68	81.76	67.13	62.93	53.39	6.21			<0.01	0.38	0.44
Milk fat: protein ratio											
Overall	1.51	1.54	1.52	1.63	1.70	0.04	0.01	0.86	<0.01	0.20	0.89
1 to 4 week	1.49	1.47	1.40	1.57	1.69	0.07			0.02	0.04	0.97
5 to 8 week	1.42	1.56	1.54	1.58	1.67	0.07			0.02	0.91	0.32
9 to 12 week	1.62	1.59	1.61	1.74	1.75	0.08			0.12	0.55	0.52

^1^ Diets with different levels of protein: CON = 15.82% of protein level; R2 = 13.85% of protein level; R4 = 11.86% of protein level; R6 = 9.84% of protein level; R8 = 7.85% of protein level. ^2^ *p*-value: T = feeding time period; R = dietary protein levels; L = linear effect of protein levels; Q = quadratic effect of protein levels; C = cubic effect of protein levels.

**Table 4:** Effects of dietary protein levels on the N metabolism of lactating goats.

Item	Diets ^1^					SEM		*p*-Value ^2^			
	CON	R2	R4	R6	R8		T	T × R	L	Q	C
N intake (g/day)											
Overall	47.07	41.62	32.95	22.09	12.60	1.70	<0.01	0.62	<0.01	0.11	0.40
1 to 4 week	41.53	36.42	26.68	17.74	11.41	2.04			<0.01	0.83	0.27
5 to 8 week	53.06	43.33	35.49	25.30	14.66	3.00			<0.01	0.71	0.81
9 to 12 week	46.61	45.11	36.68	23.23	11.73	2.99			<0.01	0.03	0.35
Fecal N excretion (g/day)											
Overall	13.98	11.54	8.55	5.57	3.10	0.50	0.06	0.42	<0.01	0.98	0.51
1 to 4 week	13.94	10.73	6.90	4.74	3.00	0.88			<0.01	0.17	0.71
5 to 8 week	15.09	11.97	8.80	6.17	3.27	0.77			<0.01	0.74	0.93
9 to 12 week	12.92	11.91	9.95	5.80	3.04	0.77			<0.01	0.05	0.34
Urinary N excretion (g/day)											
Overall	4.63	3.87	3.38	4.38	3.40	0.27	0.08	0.73	0.02	0.30	0.01
1 to 4 week	4.22	3.80	3.50	3.92	2.91	0.36			0.03	0.74	0.18
5 to 8 week	5.39	4.13	3.26	4.77	3.70	0.47			0.08	0.13	0.05
9 to 12 week	4.28	3.69	3.39	4.44	3.58	0.37			0.57	0.56	0.07
Milk N excretion (g/day)											
Overall	6.86	6.47	5.86	5.49	4.94	0.35	0.06	0.93	<0.01	0.95	0.97
1 to 4 week	6.19	5.92	5.34	5.03	4.80	0.46			0.01	0.83	0.79
5 to 8 week	7.71	6.69	6.49	5.96	4.74	0.61			<0.01	0.74	0.44
9 to 12 week	6.67	6.79	5.74	5.48	5.27	0.66			0.06	0.96	0.56
N retention (g/day)											
Overall	28.45	26.21	21.02	12.15	6.10	1.38	<0.01	0.56	<0.01	0.03	0.19
1 to 4 week	23.38	21.88	16.28	9.08	5.49	1.40			<0.01	0.28	0.09
5 to 8 week	32.59	27.23	23.43	14.36	7.69	2.30			<0.01	0.36	0.91
9 to 12 week	29.40	29.51	23.34	13.00	5.11	2.37			<0.01	0.03	0.25
Apparent Digestibility of N (%)											
Overall	69.71	71.42	73.67	73.54	74.18	1.24	0.13	0.72	0.01	0.34	0.95
1 to 4 week	66.29	70.34	73.93	72.30	73.23	1.83			0.01	0.10	0.60
5 to 8 week	71.24	71.38	74.82	73.75	75.88	1.84			0.05	0.94	0.99
9 to 12 week	71.59	72.53	72.27	74.57	73.44	1.69			0.29	0.80	0.68
Utilization efficiency of N (%)											
Overall	59.62	61.71	63.16	53.50	46.48	1.38	0.04	0.29	<0.01	<0.01	0.46
1 to 4 week	56.05	59.66	60.77	50.40	47.88	1.90			<0.01	<0.01	0.09
5 to 8 week	60.79	61.80	65.64	54.72	48.93	2.40			<0.01	<0.01	0.76
9 to 12 week	62.03	63.66	63.06	55.38	42.63	2.19			<0.01	<0.01	0.69

^1^ Diets with different levels of protein: CON = 15.82% of protein level; R2 = 13.85% of protein level; R4 = 11.86% of protein level; R6 = 9.84% of protein level; R8 = 7.85% of protein level. ^2^ *p*-value: T = feeding time period; R = dietary protein levels; L = linear effect of protein levels; Q = quadratic effect of protein levels; C = cubic effect of protein levels.

## Data Availability

The original contributions presented in this study are included in the article. Further inquiries can be directed to the corresponding author.

## Abbreviations

The following abbreviations are used in this manuscript:

ADF	Acid detergent fiber
CON	Control group, 15.82% of protein level
CP	Crude protein
DMI	Dry matter intake
FPCM	Fat and protein corrected milk
IBW	Initial average body weight
NDF	Neutral detergent fiber
OTUs	Operational taxonomic units
PCA	Principal component analysis
QIIME2	Quantitative insights into microbial ecology
R2	13.85% of protein level
R4	11.86% of protein level
R6	9.84% of protein level
R8	7.85% of protein level
